# The ecological connectivity of whale shark aggregations in the Indian Ocean: a photo-identification approach

**DOI:** 10.1098/rsos.160455

**Published:** 2016-11-16

**Authors:** Samantha Andrzejaczek, Jessica Meeuwig, David Rowat, Simon Pierce, Tim Davies, Rebecca Fisher, Mark Meekan

**Affiliations:** 1 The UWA Oceans Institute, School of Environmental Systems Engineering, University of Western Australia, Crawley, Western Australia, Australia; 2 Australian Institute of Marine Science, Crawley, Western Australia, Australia; 3 Centre for Marine Futures, Oceans Institute, University of Western Australia, Crawley, Western Australia, Australia; 4 Marine Conservation Society Seychelles, Victoria, Mahe, Seychelles; 5 Marine Megafauna Foundation, Truckee, CA 96161, USA; 6 MRAG Ltd, 18 Queen Street, London W1 J 5PN, UK

**Keywords:** citizen science, I^3^S, matching, migration, photo-ID, *Rhincodon typus*

## Abstract

Genetic and modelling studies suggest that seasonal aggregations of whale sharks (*Rhincodon typus*) at coastal sites in the tropics may be linked by migration. Here, we used photo-identification (photo-ID) data collected by both citizen scientists and researchers to assess the connectedness of five whale shark aggregation sites across the entire Indian Ocean at timescales of up to a decade. We used the semi-automated program I^3^S (Individual Interactive Identification System) to compare photographs of the unique natural marking patterns of individual whale sharks collected from aggregations at Mozambique, the Seychelles, the Maldives, Christmas Island (Australia) and Ningaloo Reef (Australia). From a total of 6519 photos, we found no evidence of connectivity of whale shark aggregations at ocean-basin scales within the time frame of the study and evidence for only limited connectivity at regional (hundreds to thousands of kilometres) scales. A male whale shark photographed in January 2010 at Mozambique was resighted eight months later in the Seychelles and was the only one of 1724 individuals in the database to be photographed at more than one site. On average, 35% of individuals were resighted at the same site in more than one year. A Monte Carlo simulation study showed that the power of this photo-ID approach to document patterns of emigration and immigration was strongly dependent on both the number of individuals identified in aggregations and the size of resident populations.

## 1. Introduction

Knowledge of the spatial extent and connectivity of populations is an essential element of the conservation strategy for any species, as it identifies the appropriate spatial context for management actions. Such information can be difficult and expensive to obtain, particularly where study species are migratory or inhabit environments where they are challenging to observe and sample. This is certainly the case for the megafauna that resides in the open ocean, including cetaceans, pinnipeds, marine reptiles, sharks and billfishes.

Whale sharks provide a good example of the issues involved with assessment of the connectivity patterns of marine megafauna. While these sharks spend the majority of their lives in the open ocean, they also form predictable seasonal aggregations of mostly juvenile males on the coastal shelves of warm temperate and tropical regions worldwide in certain locations (see Sequeira *et al*. [1] for a review). Coastal aggregations offer divers and snorkelers the opportunity to observe these placid and large animals [2–4], and the tourism industries these encounters enable now have significant economic value [5,6]. However, anthropogenic impacts on whale sharks such as hunting and ship-strike may endanger the future of both ecotourism and the species itself [7–10]. For this reason, understanding the movements, connectivity patterns and demography of populations are critical goals of management and conservation strategies for these animals.

Once departing their seasonal aggregation sites, the individual movements and ranges of whale sharks are largely unknown [11]. Genetic studies have found that populations in the Caribbean Sea appear to be distinct from all other sites, but that there is limited population structure across the Indian and Pacific ocean basins [12]. This suggests that aggregations within these two oceans are to a large extent connected, at least on an evolutionary timescale (i.e. multiple generations) [12–14]. However, theory suggests that only very low levels of genetic exchange (possibly as little as four migrants per generation) are required to homogenize alleles and produce a panmictic population [15,16]. As whale sharks are thought to have a long generation time of at least 15–37 years, rare dispersal events may be sufficient to produce an ocean-wide population with low genetic diversity [1,17], but such infrequent movements could be largely irrelevant to management of aggregations on ecological timescales.

Although genetic studies have provided the only quantitative evidence of any connection among whale shark populations via dispersal at ocean-basin scales [1], satellite telemetry has provided good evidence for connectivity at regional (hundreds to thousands of kilometres) scales [18–20]. Data from the deployment of satellite tags on whale sharks have linked aggregations in South Africa and Mozambique [21], Mozambique and Madagascar [22] and Ningaloo with both Indonesia and Christmas Island [23,24]. By contrast, evidence for broad-scale, cross-ocean movement is very limited. Potentially, this could be due to problems of the duration of tag retention on animals, because studies using satellite tags frequently report early detachment or failures in reporting data [18,23,25]. The average maximum duration of tag attachment from these listed studies describing regional movements was approximately five months. Hearn *et al*. [19] developed criteria to distinguish between tracks provided by detached, floating tags and those attached to study animals. Using these criteria, it appears unlikely that the tag reporting the longest recorded track for a whale shark of 13 000 km from the Gulf of California across the Pacific Ocean [26] was actually attached to a whale shark [19]. Hueter *et al*. [20] recorded a track of approximately 7772 km horizontal distance for a 7.5 m female whale shark over 150 days, with archived depth data revealing regular deep dives that eliminated the possibility that the tag was not attached to the animal. This broad-scale movement from the Yucatan Peninsula, Mexico to the South Atlantic Ocean was hypothesized to be for reproductive purposes [20]. Notably, the 27 other whale sharks satellite tracked in this study remained within the Caribbean Sea and Gulf of Mexico.

Theoretical models also support the possibility of broad scale (i.e. across ocean basins) movements by whale sharks. Sequeira *et al*. [1] developed a conceptual model based on data available for sightings, tracked movements and distribution that suggested that broad-scale connectivity was possible in populations of whale sharks. Their model suggested that individual whale sharks could move among the three largest ocean basins within 2–4 years and that migration from South Africa to Ningaloo was biologically plausible within 2 years. However, Sequeira *et al*. [1] concluded that although it was possible for whale sharks to move over entire ocean basins, many sharks appear to remain close to single aggregation sites for several months or years. These researchers did not speculate on the proportion of sharks within a site that might make such large migrations and the frequency with which such journeys might occur. Furthermore, they noted that the average maximum duration of attachment of satellite tags was likely to be too brief to document cross-ocean movements [1].

Here, we examined the ecological connectivity of aggregations of whale sharks across the Indian Ocean on a timescale of up to a decade. We used a photo-identification (photo-ID) approach to achieve this objective, because photographs of the stable white spot and stripe patterns that cover the dorsal surface and flanks of the body enable individual whale sharks to be uniquely identified [27–31]. By searching for matches in large libraries of photo-IDs collected at sites spread across the entire ocean basin we were able to investigate patterns in the spatial scale and frequency of movements among aggregations as well as of residency within aggregations [27–31]. One of the major issues in using our approach was the power of these analyses to detect migration given variation in sampling effort, the timing of sampling and in relative population sizes at different aggregation sites. We used a simulation study based on population sizes, resight rates and likely sampling errors to estimate the ability of our analyses to detect different levels of migration among populations across the Indian Ocean. The implications of these models for our results are discussed.

## 2. Material and methods

### 2.1 Study sites and data collection

**Figure 1.**
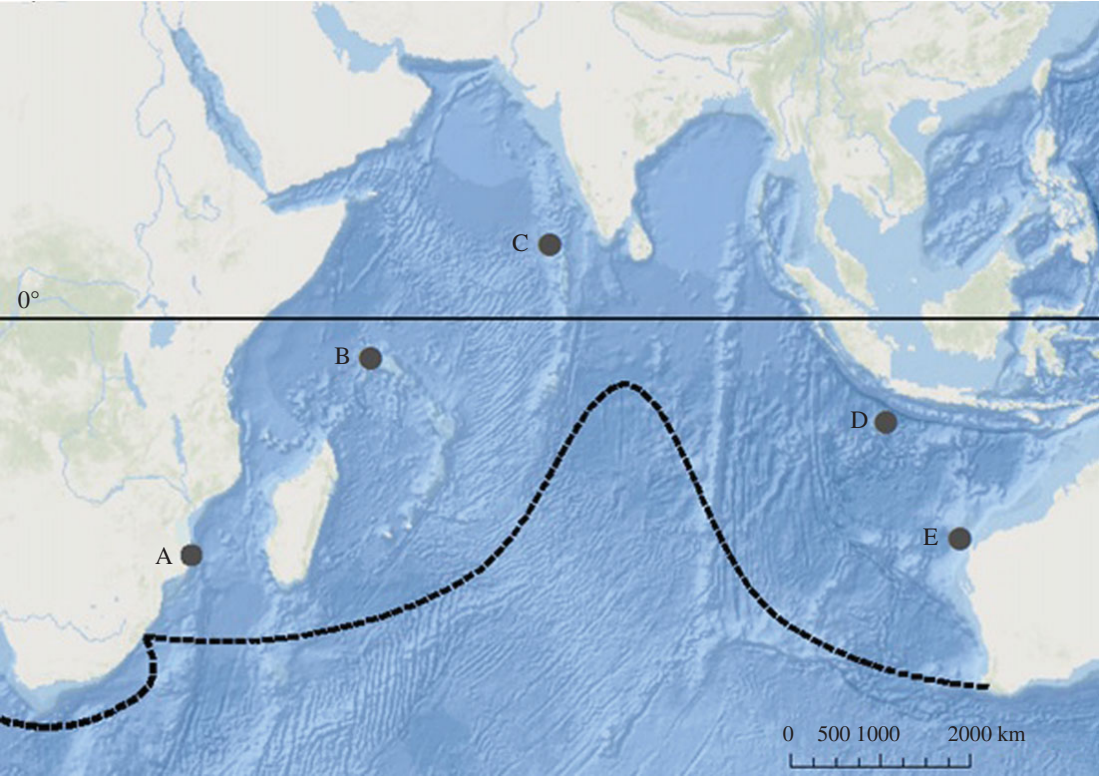
Aggregation sites for photo-ID comparisons. A, Mozambique; B, Seychelles; C, the Maldives; D, Christmas Island; E, Ningaloo Reef. Dashed black line indicates boundaries of global distribution of whale sharks. Solid black line indicates the position of the equator. (from Sequeira *et al*. [1]).

Photographs used in this study were collected by a number of research organizations, ecotourism operators and tourists from the following key aggregation sites in the Indian Ocean: Ningaloo Reef (Australia) and Christmas Island (eastern Indian Ocean); the Maldives (central Indian Ocean); and the Seychelles and southern Mozambique (western Indian Ocean; figure 1). The years for which photographs were obtained differed among sites and ranged from one (Christmas Island) to 15 years (Ningaloo; table 1). Details of photo-ID sampling at each locality can be found in the electronic supplementary material, appendix S1.

**Table 1.** The years of collection of images, the number of years images were collected (years), number of images, unique individuals identified at each site from databases that contained images of left and both flanks (LB) and right and both flanks (RB) and population estimates based on mark–recapture modelling from published studies.

site	years of collection	years	images	LB	RB	population estimate ±95% CI	study
Ningaloo	1992–1996, 2001, 2003–2011	15	3836	763	738	320–440	Meekan *et al*. [27]
Christmas Island	2007	1	73	40	35	n.d.	
Maldives	1999, 2002–2009	9	448	120	86	77–98.5	Davies *et al*. [4]
Seychelles	2003–2012	11	1221	451	405	469–557	Brooks *et al*. [28]
Mozambique	2005–2010	6	941	390	366	n.d.	
total			6519	1724	1595		

Photographs were taken by researchers, tourism operators and tourists, who swam alongside individual whale sharks and recorded the natural marks along their flanks with still or video cameras. Ideally, photos were taken on both of the right and left flanks of the shark although this was not always possible as sharks dived or swam away before both photographs could be completed. Where possible, observers recorded the sex of the shark by determining whether claspers on the pelvic fins were present (males) or absent (females) [27]. Sex was classified as indeterminate in some cases due to small size of the shark, poor visibility, or where the shark swam away before pelvic fins could be examined. Images were entered into a database and grouped according to site of capture, sex (male, female or indeterminate), and whether the photo was of the left or right flank of the whale shark.

### 2.2. Image analysis

Spot and stripe patterns present on the flanks of the shark in the area forward of the dorsal fin and behind the last gill slit were used to identify individual sharks (electronic supplementary material, figure S1). To date, no changes in these natural markings have been found to occur over time (up to 12 years) [27,30]. The public domain pattern-recognition software I^3^S (Interactive Individual Identification System) was used to look for matches of ID-photos both within and between sites [32]. Details of use of the image analysis software can be found in the electronic supplementary material, appendix S2. Image analysis was undertaken by one trained observer to minimize the effect multiple users may have had on the selection criteria for matching. Any matches were confirmed by at least one additional observer to reduce the chances of a false positive.

### 2.3. Data analysis

The total number of unique individuals photographed over the sampling period was determined in addition to the number of sightings per year for each aggregation site. The proportion of new individuals to resights in a year for each aggregation was also calculated, with resights defined as individuals recorded by photo-ID in a previous year of sampling. As Christmas Island was sampled for 1 year, photos from this site were only analysed for among-site matches and were not used in any statistical analyses.

A number of metrics were used to compare the sampled aggregations. Permutational analysis of variance (ANOVA) was used in PRIMER 6 statistical package [33] to test for differences between the average numbers of unique individuals sampled per year among sites [34]. In order to balance the design of the analysis, the database was restricted to sharks observed from 1999 to 2012. A Euclidean distance matrix with unrestricted permutation of the raw data and a total of 999 permutations were used to obtain the *p*-value, which was set at a significance level of 0.05 for all analyses. The same test was then used to identify differences between the average proportions of resights per year for each site from 1999 to 2012. For this analysis, first year of sampling was deleted from the dataset for each site, as there was no possibility of resights in that year. Lastly, permutational ANOVA was used to compare the average number of years individual whale sharks were observed at each site over the entire study period. Where significant results were found, pair-wise tests were conducted.

The number of sightings per year as a proportion of the estimated reported population was also examined. Population estimates were all derived from previous studies using Jolly–Seber open population models and included 95% confidence intervals (table 1). As Davies *et al*. [4] gave estimates derived from each of researcher and public data, the average between these two estimates was used in this comparison.

Chi-square contingency tests [35] were used to compare the number of years individual sharks returned to sites. As the minimum number of years a site was sampled was six (Mozambique), six consecutive years of data collection were used in each site; however, these years were not identical. For each site, the years included in the analysis were: Ningaloo 2006–2011; Maldives 2004–2009; Seychelles 2005–2010; and Mozambique 2005–2010. Sharks observed returning for 5 and 6 years were pooled into one group in order to meet the assumptions of *χ*^2^ that required more than 80% of cells with expected values greater than five.

As spot and stripe patterns differ between the left and right sides of a whale shark, there was a risk that single individuals could have been double-counted if each side had been photographed separately between sampling periods and/or sites [27,30]. Since the number of images for each flank was relatively similar for the Indian Ocean database, analyses were repeated for all sharks on both the left and right flanks. This meant that all sharks were considered in the analyses and the probability of identifying any migrants was maximized. Where results were of the same significance level, results were reported for the left images as these had a slightly larger sample size (table 1).

### 2.4. Sampling effort

Sampling effort at each site could not be established, because photographs were sourced from both researchers and tourists and for the latter, no effort data were available. Furthermore, techniques varied among sites, with spotter planes used on a daily basis for locating sharks at Ningaloo Reef and the Seychelles during the whale shark season, but not at Christmas Island, Maldives and Mozambique [36–38]. The database also contained photographs taken during chance encounters with whale sharks by the public and researchers.

### 2.5. Monte Carlo simulation

We used a simple Monte Carlo simulation approach to examine the sample sizes that would be required to reliably detect migration rates (MR) of varying levels among four aggregations of whale sharks (Christmas Island was excluded due to low sample sizes). The parameter we estimated was the number of unique individuals required to be observed at the sink population in a single year (*N*_sink_) given a range of MR (1–20% of the population, expressed as a proportion of the source population) and a desired detection probability (the desired certainty that a migration of a given rate would be observed) of 80 and 95%, following the equation:

$$N_{\mathrm{sink}}=\frac{\mathrm{DP}}{\left[(N_{\mathrm{source}}/P_{\mathrm{source}}\times \mathrm{MR}\times P_{\mathrm{source}})/(P_{\mathrm{sink}}+\mathrm{MR}\times P_{\mathrm{source}})\right]},$$

where *N*_source_ was the observed number of unique individuals at the source population per year; *P*_source_ was the estimated yearly size of the source population; and *P*_sink_ was the estimated yearly size of the sink population. Because of uncertainty and year-to-year variation in values of *N*_source_, *P*_source_ and *P*_sink_, these were entered into the Monte Carlo simulation by sampling a normal distribution with the appropriate mean and standard error based on the empirical data (table 1). The population estimate for the aggregation at Mozambique was based on observations from researchers based in Mozambique and the relative size of other aggregations in the western Indian Ocean.

## 3. Results

### 3.1. Stability of marking patterns

A Ningaloo whale shark photographed initially in 1992 then again in 2006 and 2011 provided strong evidence that the markings used to identify individuals remained the same over decadal time periods, with no change in patterns over 19 years (electronic supplementary material figure S2). Another 17 other individuals were also resighted at Ningaloo with at least 10 years between the first and most recent sightings. Notably, one whale shark at Ningaloo was resighted seven times over a 19 year period.

### 3.2. Whale shark abundance and sightings

**Figure 2.**
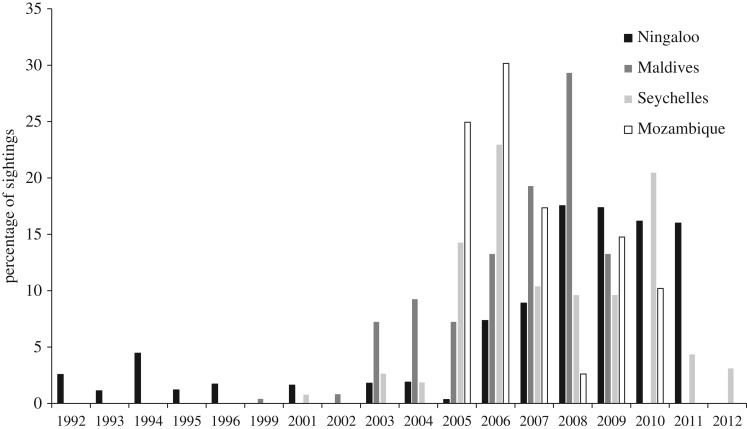
Sightings of individual sharks at each site over study period. Unless noted, all values for figures were calculated from the database of images of left flanks.

Over the 15 years of sampling, a minimum of 1724 whale sharks were recorded and uniquely identified using photo-ID (table 1). For every site, more individuals were identified using images with a combination of both flanks and the left flank only than via a combination of both flanks and the right flank only. The Ningaloo site had the highest number of uniquely identified individuals (763) as well as the largest number of years (15) sampled (table 1). Mozambique was sampled for the least number of years (6) and the Maldives had the lowest abundance of unique individuals (120). Across sites, the distribution of sightings of individuals among years varied considerably (figure 2). Overall, the highest proportion of individual sightings occurred in 2006. At Ningaloo, sightings were low prior to 2007 and increased substantially after this time. In the Maldives, sightings peaked in 2008 while in the Seychelles, peaks in sightings occurred in 2006 and 2010. In Mozambique, the largest proportion of sightings occurred in 2005 and 2006 (figure 2).

**Figure 3.**
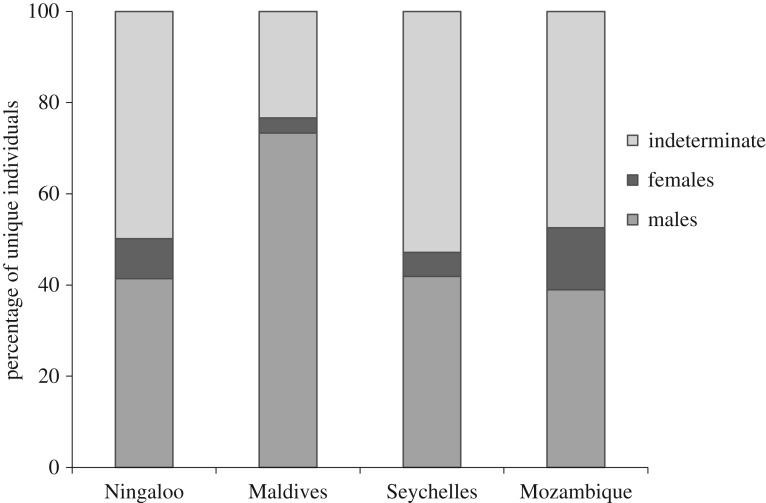
Composition of sampled populations at each site by sex (male, female and indeterminate) from 1999 to 2012 for sharks with left and both flank images.

At all sites, there was a much higher proportion of male whale sharks relative to females (figure 3). Overall, whale sharks classified as indeterminate sex outnumbered those for whom sex had been identified. The aggregation at Mozambique had the lowest proportion of males (39.0%), while the Maldives had the highest (73.3%; figure 3). The Maldives also had the lowest proportion of female whale sharks (3.3%) and sharks of indeterminate sex (23.3%). The Seychelles had the highest proportion of indeterminate sharks (52.9%), and Mozambique had the highest proportion of female whale sharks (13.6%).

The average yearly abundance of photographed individuals differed significantly among sites (*F*_3,32_ = 2.979, *p *= 0.042; electronic supplementary material, figure S3), with pair-wise tests showing that abundance was greater at both Ningaloo and Mozambique than the Maldives (*p* = 0.02 for each). Ningaloo had the highest average yearly abundance of sampled individuals (104), followed by Mozambique (77), the Seychelles (59) and the Maldives (28) (electronic supplementary material, figure S3).

### 3.3. Resightings relative to estimated population size

Population size and resights were negatively correlated, so that aggregation sites with relatively large population sizes had lower rates of yearly resights. In the Maldives, 32% of the population of 88 individuals was resighted each year, while at Ningaloo 20% of the population of 380 animals was resighted and in the Seychelles only 10.6% of the estimated population of 513 individuals was resighted each year.

### 3.4. Resights within aggregation sites

**Figure 4.**
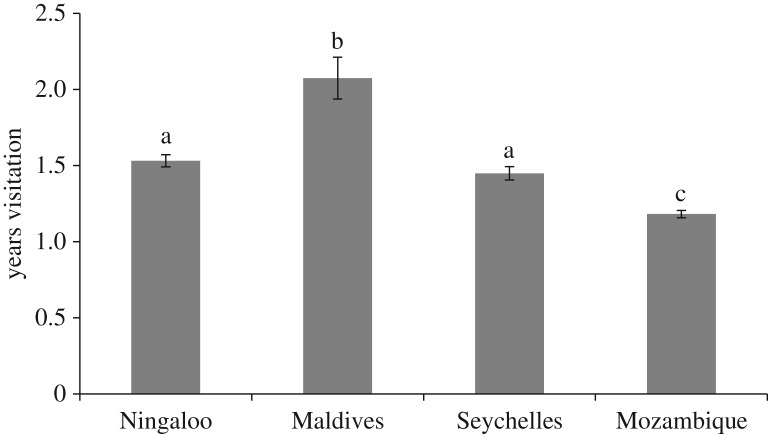
The mean number of years (±s.e.) that individual whale sharks were observed within each site. Letters indicate sites that were significantly different.

**Figure 5.**
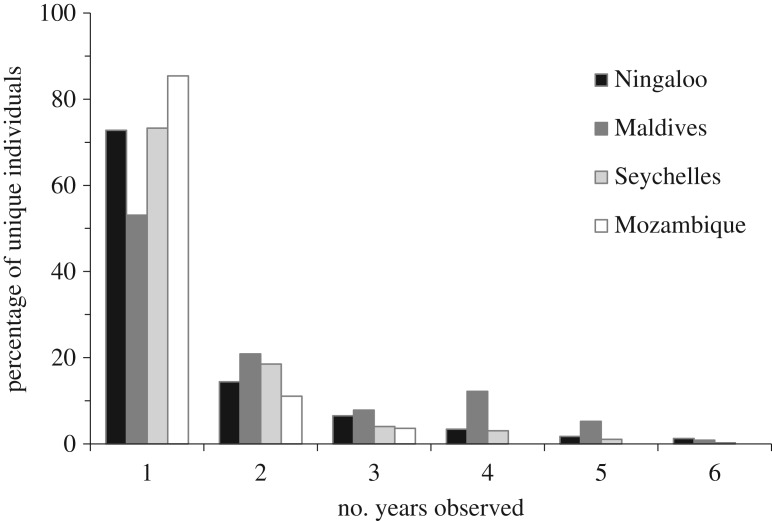
The percentage of individuals observed within a region over six successive years of sampling: Ningaloo (2006–2011), Maldives (2004–2009), Seychelles (2005–2010) and Mozambique (2005–2010).

The average number of years individual whale sharks were observed visiting a site differed significantly among sites (*F*_3,1714_ = 27.02, *p* < 0.0001) (figure 4). Pair-wise tests showed that only Ningaloo and the Seychelles did not differ in rates of resighting (*p *= 0.19; *p* < 0.001 in all other combinations of sites). The Maldives had the highest average resighting rate of 2.08 ± 0.14 years and Mozambique the lowest at 1.18 ± 0.02 years (figure 4). The distribution of the number of years individual whale sharks returned also differed significantly among sites (*χ*^2^_12, 0.001_ = 32.91, *χ*^2^ cal = 95.00, *p *< 0.001; figure 5). Overall, nearly two-thirds of whale sharks were observed in only 1 year at each site, with the remainder (35%) resighted at least once. Over the 6 years of sampling used in the analysis, a small proportion (less than 2%) of individuals at Ningaloo, the Maldives and the Seychelles were observed in all 6 years. At Ningaloo, 72.8% of individuals were only observed in 1 year, and 14.4% in 2 years, with resights further declining with each additional year of sampling. The Maldives had the lowest proportion of individuals observed in only 1 year (53%); 20.9% of individuals were observed in 2 years and unexpectedly, more were resighted in 4 years than 3 years (12.2 and 7.9%. respectively). Resights of whale sharks in the Seychelles followed the same general pattern as at Ningaloo Reef. Mozambique had the lowest levels of resights with 85.4% of individuals observed in only 1 year (figure 5). Additionally, three years was the maximum number of years an individual shark was resighted in this aggregation, the shortest maximum resighting period across all sites. Over the entire sampling period, the maximum number of consecutive years a whale shark was resighted at an aggregation was seven, which occurred at both the Seychelles and Ningaloo sites.

On average, the proportion of resightings to new individuals within a year of sampling did not differ significantly among sites (*F*_3,29_ = 1.82, *p *= 0.18). All sites followed similar general patterns of a consistent proportion of resights to new individuals after the first few years of sampling (electronic supplementary material, figure S4). The Maldives had the highest proportion of resights relative to new individuals in the later years of sampling.

### 3.5. Connectivity among sites

Only one of the 1724 individuals in the database was resighted at a different aggregation. This male whale shark was observed at the site off Mozambique in November 2005 and late January 2010 and then again in the Seychelles in September 2010. This whale shark had travelled a minimum straight line distance of 3000 km over 221 days at a minimum swimming speed of 13.6 km d^−1^ to complete the journey between Mozambique and the Seychelles.

### 3.6. Monte Carlo simulation

**Figure 6.**
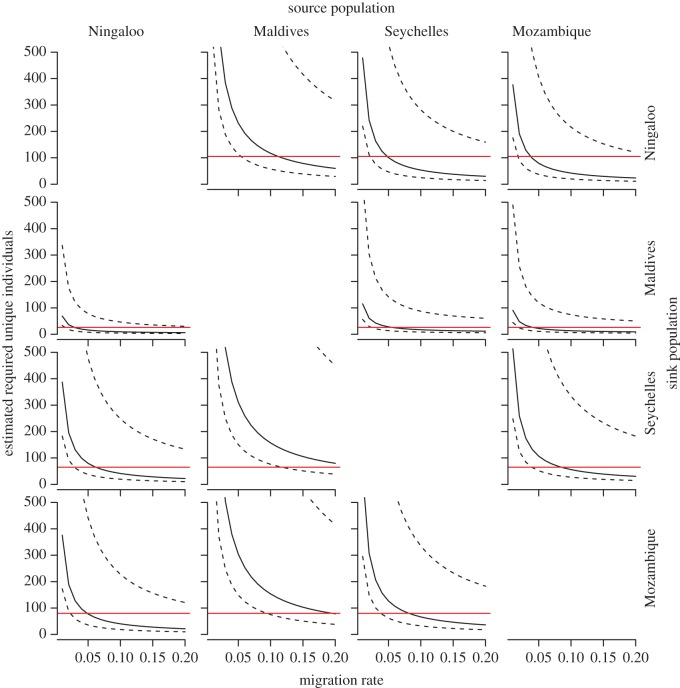
Plot of number of whale sharks required to be identified per year to have an 80% chance of sighting a migrant given different rates of migration between source and sink populations in the Indian Ocean. Solid black lines represent the median (50th percentile) values obtained from the Monte Carlo simulation, with the upper and lower dashed lines representing the 2.5th and 97.5th percentiles values, respectively, thereby approximating 95% confidence bands. Solid red line shows the average number individuals identified in the sink population in any one year.

Our simulation showed that we had better chances of detecting migrants (if they were present) to sink populations at Ningaloo Reef and the Maldives than the Seychelles and Mozambique given current numbers of individuals identified each year (figure 6). At Ningaloo, a migration rate of at least 12–13% could be detected from the Maldives, and approximately 5% per year from source populations in the Seychelles and Mozambique. In the Maldives, we had 80% confidence of detecting migrants at immigration rates of at least 2–5% from all three other source populations (figure 6). In comparison, the simulation shows that immigrants into the Seychelles and Mozambique aggregations would be more difficult to detect with 80% confidence. For these sites, MR as high as 20% per year might not be detected given current rates of identification (figure 6). On average, an immigration rate of 10% of sharks from Mozambique into the Seychelles site would have an 80% chance of being detected. Notably, immigrants from Ningaloo Reef would be detected at the lowest MR for each sink population when compared with other potential source sites.

## 4. Discussion

### 4.1. Connectivity of Indian Ocean aggregations

No evidence of cross-ocean migration by individual whale sharks was found by our comparison of photo-ID databases from sites spread across the Indian Ocean. However, a regional connection was confirmed. A male shark photographed on the coast of Mozambique in January 2010 and in the Seychelles in September of the same year provided evidence of an ecological connection between these two aggregation sites. This is the second observation of movement between these sites; the first was a shark tagged in the Seychelles in November 1996 that was resighted 11 months later in Mozambique [2]. In our study, the shark matched by photo-ID must have swum a minimum of 13.6 km d^−1^ in order to reach the Seychelles eight months after it was photographed in Mozambique. This value is well within the average swimming speeds recorded by earlier studies of 30 km d^−1^ [1], implying either that the animal did not take a direct route between these sites or was moving at less than average speeds.

Our evidence for limited dispersal must, however, be considered in the context of our simulation studies that examined the power of the photo-ID technique to detect MR. These simulations showed that we had a relatively high chance of detecting migrants to sink populations in the Maldives and at Ningaloo Reef. In the case of the former, the resident population size was very small (only 80–100 sharks), while in the latter our database of IDs was very large (at least 738 uniquely identified sharks; table 1). Conversely, the database of IDs in both the Seychelles and Mozambique was smaller relative to the likely size of resident populations, with models suggesting that we had relatively low power to detect migration from other sources to these sink populations. Despite this problem, it is notable that we were able to detect a migrant moving between the Seychelles and Mozambique. If our simulation studies are indeed representative, it seems possible that rates of migration between these sites may be relatively high, with at least 10% of individuals per year (or more) moving between locations. Overall, our simulations suggest that given present sample sizes, effort and rates of resighting, the power of the photo-ID approach to detect low (less than 5%) rates of migration among localities is relatively weak, except where resident populations are very small, as is the case in the Maldives. It is also important to note that our simulation generated large confidence intervals around estimates of medians, suggesting that variation in factors such as sampling effort and environmental conditions could alter the potential for resighting.

The lack of evidence for movements at ocean-basin scales in photo-ID records combined with the results of our Monte Carlo simulation suggests that either these are likely to be rare events, or that they are occurring in parts of the population (such as mature animals) that are not present at aggregation sites and are thus inaccessible to the photo-ID approach. So where do these juvenile males go when they depart their seasonal aggregations? Similar to our study, satellite tagging studies have revealed evidence for migrations of whale sharks at regional scales, both in the Indian Ocean and elsewhere. For example, Brunnschweiler *et al*. [22] tracked a shark off the coast of Mozambique in February 2006 to the southern tip of Madagascar over a period of three months. Both photo-ID and conventional and satellite tags were used by Hueter *et al*. [20] to record movements of individual whale sharks that formed a summer feeding aggregation off the northeastern Yucatan Peninsula, Mexico into other regions of the Gulf of Mexico, the northwestern Caribbean Sea and the Straits of Florida. In the Galapagos Islands, a whale shark was tracked by a satellite tag to a distance 1650 km to the west before returning to the Islands four months later [19]. At Ningaloo Reef, movements of six whale sharks were tracked using satellite tags for up to seven months and these animals travelled a maximum straight line distance of 1500 km to the northeast [23]. More recently at the Ningaloo Reef aggregation, a combined satellite tracking, photo-ID and public survey approach revealed an extended distribution of sharks along the Western Australian coastline, with sightings in this region observed in all months of the calendar year [39]. Despite these results, identification of migration patterns at annual scales within this region remains incomplete, largely because of the inability of researchers to deploy tags on sharks for more than six to eight months. It is also possible that some juvenile males do not depart Ningaloo Reef and may instead maintain year-round residence, but are simply using deeper habitats in the seasons when they are assumed to be absent. For example, Cagua *et al*. [40] compared photo-ID and passive acoustic telemetry data of sharks at an aggregation site off the coast of Tanzania. Results from photo-ID displayed a clear seasonal pattern of sightings, with no whale sharks observed between March and September, whereas acoustic data demonstrated a year-round residence of the same animals, with sharks swimming deeper and further away from shore during this time and thus being inaccessible to data collection using photo-ID [40]. It is also possible that such clandestine behaviour may mask movements at larger spatial scales rather than just within aggregation sites.

In contrast to the results of our photo-ID comparison, genetic studies suggest that whale sharks across the Indian Ocean form a single, panmictic population [12]. Our simulation showed that low, but potentially significant (at least in terms of gene flow) rates of long-distance dispersal at ocean-basin scales are unlikely to be detected by the photo-ID technique. The weight of evidence from both tagging and our photo-ID study does suggest, however, that regional movements may be far more common than movement at large scales. If this is the case, panmixis can still occur in populations through a form of genetic ‘hopscotch’, whereby sharks breed with others in neighbouring regions, and their offspring subsequently move to more distant areas [14]. Alternatively, juvenile males may not be representative of the rest of the population and the long-distance movements of mature sharks may be responsible for the lack of genetic structure of populations across the Indian Ocean. Generally, home range size and reported satellite tracks increase with shark size, with some of the longest whale shark tracks to date coming from mature females [20,41,42]. Only a small number of these long-distance migrations and subsequent breeding would need to occur in order to produce panmixis [16]. If these movements were rare, they might be largely irrelevant to the management of aggregation sites on ecological (as opposed to genetic) timescales.

### 4.2. Future directions and management implications

Photo-ID techniques may be useful for tracking the movement patterns of a large sample size of whale sharks at regional spatial scales, in which case the data obtained from the approach could be improved by more intensive sampling at this scale within the Indian Ocean [1], for example, along the coastline of eastern Africa. An expansion of photo-ID sampling efforts in the region of Southeast Asia would also greatly benefit our understanding of regional movements. Here, a whale shark photographed in Taiwan in 2012 was resighted in the Philippines in 2013 [43]. A combined satellite telemetry and photo-ID approach might also be valuable, as has shown to be the case for whale sharks that aggregate seasonally off the Yucatan Peninsula [20] and Ningaloo Reef [39] and for other species such as white sharks (*Carcharodon carcharias*) in South Africa [44], and would likely enhance our understanding of whale shark movements in the Indian Ocean.

Although whale sharks and white sharks occupy very distinct ecological niches, photo-ID methods have been applied for both species and demonstrated similar patterns of site fidelity. This demonstrates the potential applicability of photo-ID approaches for other elasmobranchs that possess body patterns allowing them to be individually identifiable [30]. Standardization of methods and variables recorded when sampling would allow for cross-species comparison of site fidelity and movement patterns.

Regional patterns of connectivity of whale shark aggregations suggests that conservation and management approaches should focus at this scale [45,46]. Cross-jurisdictional management will be required, although to a much lesser degree and involving far less complexity than would be the case if cross-ocean movements were commonplace. Even at this smaller scale, regional approaches to management are probably to face substantial hurdles, given the track record of initiatives such as the management of fish stocks that migrate through, or occur in, more than one exclusive economic zone [47]. These are known as straddling stocks and the catch by one country can influence the fisheries of others [48]. According to international law, these stocks should be managed cooperatively through regional fisheries management organizations (RFMOs). Such cooperation and enforcement can be very difficult to achieve, especially as membership to RFMOs is voluntary and non-members can undermine cooperative efforts [47,48]. An understanding of the successes and challenges faced by RFMOs could be used to assist in the development of regional management and conservation plans for whale sharks.

### 4.3. Limitations of photo-identification and the effect of population size

The semi-automated process of photo-ID has the potential to match two unique individuals (false positive) or fail to find a match even though it does exist (false negative). By examining matches by eye, we reduced the possibility of a false positive and previous studies have found I^3^S to be a useful tool in minimizing the rate of false negatives [49]. The use of only one trained observer in image analysis further reduced the chances of a false negative by minimizing the variation in subjectivity when fingerprinting images. Although a majority of individuals had photo-ID images of both sides of their flanks, there was still the possibility that a match may have been missed as opposite sides of the shark could have been photographed in different locations. The growing size of photo-ID databases and resights within locations will further reduce the likelihood of this event.

A lack of records of sampling effort for each site also hindered the interpretation of data. Our findings of year-to-year variation in abundance and resight rates were most likely due to differences in sampling effort. Citizen science programmes could assist in this regard by reporting measures of effort such as number of days sampled per season and number of whale shark encounters per trip. However, such data will always be complicated by the variation in techniques (such as the use of spotter planes to locate sharks) among sites.

Although all four aggregations displayed similar proportions of new and resighted sharks, the numbers of years that individual sharks were resighted differed significantly among aggregations. This was probably due to the varying population sizes of the aggregations, so that in the Maldives, where the population was only around 90 sharks, the greatest proportion of the population was sampled on a year to year basis. Consequently, there was an enhanced probability of resighting resident sharks in successive years and new migrants. In the Seychelles, where population size was much larger, a relatively low proportion of the population was sampled and there was thus less chance of returning whale sharks being resighted or the identification of migrants from other source populations. The large confidence limits around the curves produced by our simulation reflect the impact this type of variability had on the chances of identifying migrants.

### 4.4. Conclusion

Despite the results of genetic studies, any evidence for the cross-ocean connectivity of whale shark aggregations on ecological timescales remains elusive. Our study suggests that photo-ID techniques are unlikely to be of use for the understanding of movement patterns at such scales because these broad-scale movements, if they occur, are likely to be relatively rare events that we may not have the power to detect. At smaller, regional scales, photo-ID studies may be of greater use but considerable effort will be required to build libraries of identifications in order to detect movement patterns where population sizes are relatively large. Moreover, better coverage of sampling sites at regional scales will also aid understanding of the frequency, extent and degree of residency of whale sharks at aggregation sites.

## Supplementary Material

Appendix S1. Photo-ID sampling methods Describes photo-ID data collection for each aggregation site

## Supplementary Material

Appendix S2. The use of image analysis software Describes the use of I3S in the photo-ID analysis of whale sharks

## Supplementary Material

Appendix S3. Indian Ocean whale shark database

## Supplementary Material

Figure S1. Fingerprinted standardised reference area of a whale shark Demonstrates how the whale shark images were fingerprinted to standardise the area of comparison in image analysis

## Supplementary Material

Figure S2. Whale shark re-sighted over a 19 year period at Ningaloo Two images from the same whale shark 19 years apart to demonstrate unchanging spot patterns

## Supplementary Material

Figure S3. Average yearly abundance of whale sharks at each site Differences in average yearly abundance of photographed individuals at each site

## Supplementary Material

Figure S4. The number of resights and new individuals per year at each site Proportion of resights to new individuals at each site

## Data accessibility

All data are provided in the electronic supplementary material.
