# Supplementary material for: The ecological connectivity of whale shark aggregations in the Indian Ocean: a photo-identification approach
Source: R Soc Open Sci. 2016 Nov 16;3(11):160455. doi: 10.1098/rsos.160455 (PMC5180127; doi:10.1098/rsos.160455)
Supplement: Appendix S1. Photo-ID sampling methods Describes photo-ID data collection for each aggregation site [file rsos160455supp1.docx]

Supplementary 1. Description of photo-ID data collection at each aggregation site.

Due to the seasonality of aggregations, the months that data were typically collected at each site also differed. At both Ningaloo and the Seychelles, spotter planes were used to locate whale sharks and direct tourist and research boats to their vicinity. Snorkelers then entered the water to collect photo-ID images. At the Ningaloo aggregation, tourist boats based at Tantabiddi and Coral Bay tended to operate within 2-3 km seaward of the reef edge within the Ningaloo Marine Park between March and July. In the Seychelles, whale shark tourist boats focused around the coastal zone of the island of Mahe from July to November, with operations extending to a maximum of four kilometres offshore [1]. At Christmas Island, research boats operated in the nearshore waters of the island in January, searching for sharks along the edge of the reef drop-off [2]. In the Maldives, the majority of data were collected from two whale shark encounter hotspots: Maamiogili-Dhigurah Reef in the South Ari Atoll, and Hanifaru Lagoon in Baa Atoll, from April to June each year [3]. In Mozambique, tourist and research boats operated from Tofo Beach year-round, with raised spotting chairs on boats used to assist in searching for sharks [4]. Search effort in this area focuses on an eight kilometre strip of coastline south of Tofo, operating up to one kilometre offshore [5]. All five aggregations have been reported to be dominated by juvenile males [2, 6-9].

1 Rowat, D., Speed, C. W., Meekan, M. G., Gore, M. A., Bradshaw, C. J. A. 2009 Population abundance and apparent survival of the Vulnerable whale shark *Rhincodon typus* in the Seychelles aggregation. *Oryx*. **43**, 591-598. (doi:10.1017/S0030605309990408)

2 Meekan, M. G., Jarman, S. N., McLean, C., Schultz, M. B. 2009 DNA evidence of whale sharks (*Rhincodon typus*) feeding on red crab (*Gecarcoidea natalis*) larvae at Christmas Island, Australia. *Marine and Freshwater Research*. **60**, 607-609. (10.1071/mf08254)

3 Davies, T. K., Stevens, G., Meekan, M. G., Struve, J., Rowcliffe, J. M. 2012 Can citizen science monitor whale-shark aggregations? Investigating bias in mark–recapture modelling using identification photographs sourced from the public. *Wildlife Research*. **39**, 696-704. (<http://dx.doi.org/10.1071/WR12092>)

4 Pierce, S. J., Méndez-Jiménez, A., Collins, K., Rosero-Caicedo, M., monadjem, A. 2010 Developing a Code of Conduct for whale shark interactions in Mozambique. *Aquatic Conservation: Marine and Freshwater Ecosystems*. **20**, 782-788. (10.1002/aqc.1149)

5 Rohner, C. A., Pierce, S. J., Marshall, A. D., Weeks, S. J., Bennett, M. B., Richardson, A. J. 2013 Trends in sightings and environmental influences on a coastal aggregation of manta rays and whale sharks. *Marine Ecology Progress Series*. **482**, 153-168. (10.3354/meps10290)

6 Bradshaw, C. J. A., Mollet, H. F., Meekan, M. G. 2007 Inferring population trends for the world's largest fish from mark-recapture estimates of survival. *Journal of Animal Ecology*. **76**, 480-489. (10.1111/j.1365-2656.2006.01201.x)

7 Brooks, K., Rowat, D., Pierce, S. J., Jouannet, D., Vely, M. 2010 Seeing spots: photo-identification as a regional tool for whale shark identification. *Western Indian Ocean Journal of Marine Science*. **9**, 185-194.

8 Riley, M. J., Hale, M. S., Harman, A., Rees, R. 2010 Analysis of whale shark *Rhincodon typus* aggregations near South Ari Atoll, Maldives Archipelago. *Aquatic Biology*. **8**, 145-150. (10.3354/ab00215)

9 Rohner, C. A., Richardson, A. J., Prebble, C. E. M., Marshall, A. D., Bennett, M. B., Weeks, S. J., Cliff, G., Wintner, S. P., Pierce, S. J. 2015 Laser photogrammetry improves size and demographic estimates for whale sharks. *PeerJ*. **3**, e886. (10.7717/peerj.886)
