# Supplementary material for: The ecological connectivity of whale shark aggregations in the Indian Ocean: a photo-identification approach
Source: R Soc Open Sci. 2016 Nov 16;3(11):160455. doi: 10.1098/rsos.160455 (PMC5180127; doi:10.1098/rsos.160455)
Supplement: Appendix S2. The use of image analysis software Describes the use of I3S in the photo-ID analysis of whale sharks [file rsos160455supp2.docx]

Supplementary 2. Description of the use of I3S in the photo-ID analysis of whale sharks.

Each image was initially ‘fingerprinted’ with its unique spot pattern in a standardised reference area for comparison (Fig. SF1). For this study, the standardised area was defined by a) the top of the 5th gill slit, b) the bottom of the 5th gill slit and (c) the posterior-most point where the pectoral fin reaches the body. This area was chosen as it was easy to identify, consistent with previous studies, and it appeared in a large majority of the photographs [1, 2]. As the number of spots defined on each fingerprint may affect the I^3^S matching process, only 12 spots were placed by the observer within the reference area of each photo. The most prominent spots within the reference area were chosen for analysis. In a case where 12 spots were not present within the reference area, spots closest to the defined area were used.

Comparisons were firstly made of fingerprinted images of sharks within sites. Images from each site were added to the I^3^S database separately to be analysed. Images were compared one by one to similarly fingerprinted images within the database using a 2D linear algorithm. This algorithm calculated the position of each marked spot relative to the reference area and compared the position of each marked spot with its potential ‘pair’ on each image in the database using linear transformation [3]. The resulting output gave scores indicating the quality of a match between images of two sharks and was ranked with decreasing likelihood of an actual match. The top 20 possible matches for each shark were examined by eye to confirm resights and the years the individual shark was observed were recorded. When image quality was too poor for matching, photographs were discarded from the database. Once all images had been matched within each site, they were then compared with those from the image databases of other sites using the same protocol.

1 Speed, C. W., Meekan, M. G., Bradshaw, C. J. A. 2007 Spot the match–wildlife photo-identification using information theory. *Frontiers in zoology*. **4**, 1-11. (10.1186/1742-9994-4-2)

2 Meekan, M. G., Bradshaw, C. J. A., Press, M., McLean, C., Richards, A., Quasnichka, S., Taylor, J. G. 2006 Population size and structure of whale sharks *Rhincodon typus* at Ningaloo Reef, Western Australia. *Marine Ecology Progress Series*. **319**, 275-285. (10.3354/meps319275)

3 Brooks, K., Rowat, D., Pierce, S. J., Jouannet, D., Vely, M. 2010 Seeing spots: photo-identification as a regional tool for whale shark identification. *Western Indian Ocean Journal of Marine Science*. **9**, 185-194.
