# Supplementary material for: The ecological connectivity of whale shark aggregations in the Indian Ocean: a photo-identification approach
Source: R Soc Open Sci. 2016 Nov 16;3(11):160455. doi: 10.1098/rsos.160455 (PMC5180127; doi:10.1098/rsos.160455)

Figure S1. Left flank of a whale shark fingerprinted in I^3^S. The 12 most prominent spots were marked with a dot. Lines and inconspicuous marks were avoided. The standardised reference area was marked by the top and bottom of the 5^th^ gill slit and the posterior-most point where the pectoral fin meets the body.


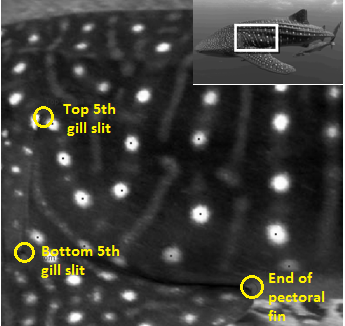

Supplement: Figure S1. Fingerprinted standardised reference area of a whale shark Demonstrates how the whale shark images were fingerprinted to standardise the area of comparison in image analysis [file rsos160455supp4.docx]
