# Supplementary figures and images for: The ecological connectivity of whale shark aggregations in the Indian Ocean: a photo-identification approach
Source: R Soc Open Sci. 2016 Nov 16;3(11):160455. doi: 10.1098/rsos.160455 (PMC5180127; doi:10.1098/rsos.160455)

Figure S2. A whale shark first sighted at Ningaloo in 1992 (upper photo) and again in 2011 (lower photo).


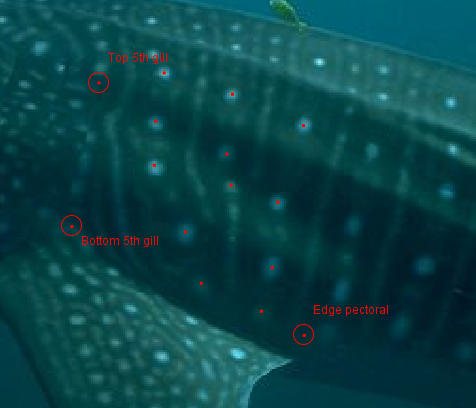

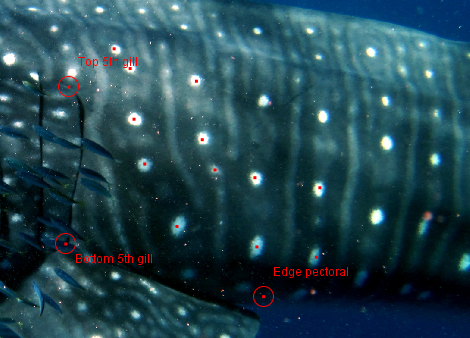

Supplement: Figure S2. Whale shark re-sighted over a 19 year period at Ningaloo Two images from the same whale shark 19 years apart to demonstrate unchanging spot patterns [file rsos160455supp5.docx]
