# Supplementary material for: The ecological connectivity of whale shark aggregations in the Indian Ocean: a photo-identification approach
Source: R Soc Open Sci. 2016 Nov 16;3(11):160455. doi: 10.1098/rsos.160455 (PMC5180127; doi:10.1098/rsos.160455)
Supplement: Figure S3. Average yearly abundance of whale sharks at each site Differences in average yearly abundance of photographed individuals at each site [file rsos160455supp6.docx]

Figure S3. The average mean yearly abundance of unique individuals at each site from 1999-2012 ± standard errors. Dark grey bars are based on observations from sharks with images from left and both flanks; light grey bars are based on observations from sharks with images from right and both flanks. Same letters indicate sites of no significant difference.
