# Supplementary material for: The ecological connectivity of whale shark aggregations in the Indian Ocean: a photo-identification approach
Source: R Soc Open Sci. 2016 Nov 16;3(11):160455. doi: 10.1098/rsos.160455 (PMC5180127; doi:10.1098/rsos.160455)
Supplement: Figure S4. The number of resights and new individuals per year at each site Proportion of resights to new individuals at each site [file rsos160455supp7.docx]

Figure S4. The number of resights (hatched bar) and new individuals (solid bar) per year at A) Ningaloo, B) Maldives, C) Seychelles, and D) Mozambique from 2001-2012.


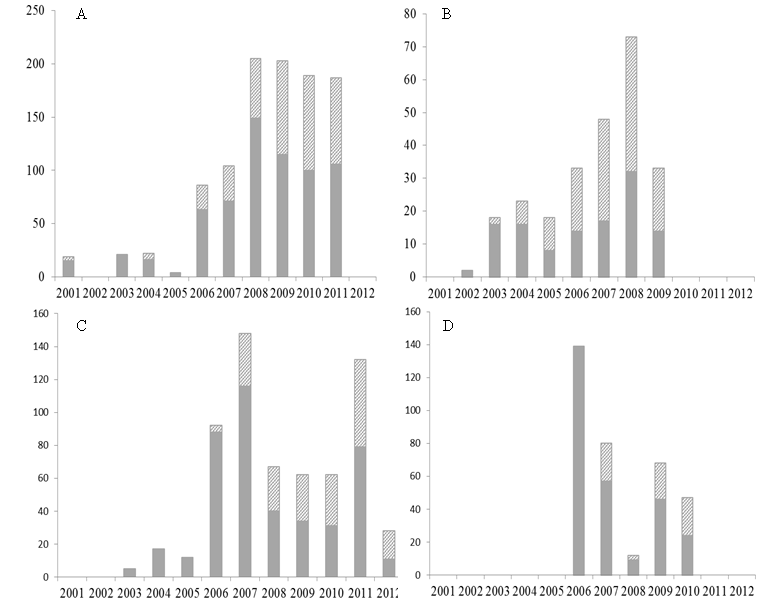


Number of Individuals
